# The effects of community-based exercise modalities and volume on musculoskeletal health and functions in elderly people

**DOI:** 10.3389/fphys.2023.1227502

**Published:** 2023-07-10

**Authors:** Chin Leong Lim, Nicholas Ling Swee Keong, Margaret Mei Chan Yap, Alvin Wai Kit Tan, Cher Heng Tan, Wee Shiong Lim

**Affiliations:** ^1^ Lee Kong Chian School of Medicine, Nanyang Technological University, Singapore, Singapore; ^2^ Department of Endocrinology, Tan Tock Seng Hospital, National Healthcare Group, Singapore, Singapore; ^3^ Department of Diagnostic Radiology, Tan Tock Seng Hospital, National Healthcare Group, Singapore, Singapore; ^4^ Department of Geriatric Medicine, Institute of Geriatrics and Active Aging, Tan Tock Seng Hospital, Singapore, Singapore

**Keywords:** muscle loading exercise, muscular and bone health, body composition, bone mineral density, flexibility, balance, strength

## Abstract

The effects of different muscle loading exercise (MLEX) modes and volume on musculoskeletal health is not well-studied in older populations.

**Aim:** Therefore, this study aimed to compare the effects of community-based MLEX modalities and volume on musculoskeletal health in elderly people.

**Methods:** Elderly men (n = 86) and women (n = 170), age 50–82 years old, were assigned to the sedentary (SE, n = 60), muscle strengthening exercise (MSE, n = 71), aerobic exercise (AE, n = 62) and Tai Chi exercise (TCE, n = 63) groups, based on > 2 years of exercise history. Exercise volume was compared between “Minimum” (“Min” < 60 min/week), “Low” (60–120 min/week). “Moderate” (121–239 min/week) and “High” (240–720 min/week) volumes.

**Results:** All three modes of MLEX were associated with lower percentage of body fat (BF%) and higher percentage of lean body mass (LBM%, *p* = 0.003 main effect of group, and *p* = 0.002 main effect of volume for both BF% and LBM%), but not with higher bone mineral density (BMD, total body, lumbar spine, total hip and neck of femur), than SE. TCE had a distinct advantage in trunk flexibility (*p* = 0.007 with MSE, *p* = 0.02 with AE, and *p* = 0.01 with SE), and both TCE (*p* = 0.03) and AE (*p* = 0.03) performed better than SE in the one-leg stand balance test. Isometric strength and throwing speed and peak power with a 2 kg power ball were higher in the MLEX than SE groups (*p* = 0.01), in the ranking order of MSE, AE and TCE. However, there was no difference in handgrip strength performance between the MLEX groups, which performed better than the SE participants. Accumulating >120 min/week of MLEX can promote body composition health and muscle functions, but 60 min/week of MSE alone may have equal or better outcomes in these parameters.

**Conclusion:** Community-based MLEX classes may be used to mitigate age-related chronic disease that are associated with body composition and muscular functions.

## 1 Introduction

The musculoskeletal (MSK) system plays major roles in human mobility and functions over lifespan, and the ability to perform physical work is associated with the state of muscle mass and functions (MMF) (Cruz-Jentof et al., 2010; Lavin et al., 2019; Bull et al., 2020). Within limits of load tolerance, the MSK system is highly responsive to the magnitude and volume of load exposure (Doherty, 2003; Beck, 2022). Frequent load exposure increases BMD and MMF, and prolonged absence of load exposure reverses these load-induced adaptations (Staron et al., 1991; Suzuki et al., 1996; Doherty, 2003; Martin et al., 2017; Kitsuda et al., 2021; Abilgaard et al., 2022). For example, BMD of young swimmers, a non-load bearing sport, was negatively correlated with the number of years of participation (Agostinete et al., 2020) and BMD of adolescent footballers, a load bearing sport, was higher by 7%–14% than swimmers, cyclists, and sedentary individuals (Vlachopoulos et al., 2017). Lower body strength and cross-sectional area of fast twitch muscle fibres were also closely associated with training load profiles over 20 weeks of strength training, followed by 30–32 weeks of detraining, and another 6 weeks of retraining (Staron et al., 1991). This evidence demonstrates the positive association between load exposure, BMD and MMF and supports the notion that muscle loading exercise (MLEX) can potentially mitigate age-related decline in MSK health.

Ageing is associated with decreased BMD and MMF (Frontera et al., 2000; Go et al., 2013; Rondanelli et al., 2014) and an increase in the prevalence of osteoporosis (Ahlborg et al., 2010; Goh and Hart, 2016) and sarcopenia (Akune et al., 2014; Cruz-Jentoft et al., 2014). The prevalence of osteoporosis increased by 10-fold in women and by 8-fold in men between 50 and 80 years of age (Woolf and Pfleger, 2003). The prevalence of sarcopenia among the elderly varies widely between 1%–33%, depending on the region and community of interest. Compared with younger adults, maximum strength in older adults, 65–90 years old, was lower by 20%–50% (Frontera et al., 1991; Doherty, 2003). Muscular strength in the elderly decreased by 20%–30% over 12 years, which corresponded with a 14.7%–16% decrease in muscle cross-sectional area that accounted for 90% of strength loss (Frontera et al., 2000). The decline of the MSK system with age has been attributed to multiple factors, such as changes in the neuro-endocrine systems, diet, and lower physical activity levels (Jones et al., 2009; Kalyani et al., 2014; Hygum et al., 2017; Lavin et al., 2019; Noh et al., 2020; Rozand et al., 2020; Chandra and Rajawat, 2021; El-Gazzar and Högler, 2021). However, there is strong evidence suggesting that MLEX is effective for mitigating age-related decline in MSK health (Frontera et al., 1988; Jones et al., 2009; Mezil et al., 2015; Dent et al., 2017; Lavin et al., 2019). This evidence forms the bases for the current consensus to promote weekly MLEX in young and older populations, including those with chronic disease (Garber et al., 2011; Thornton et al., 2016; Bull et al., 2020).

The protective properties of MLEX on MSK health was shown in a meta-analysis involving >200 osteoporosis and osteopenia patients. BMD in the lumbar spine was significantly higher in these patients than control group patients after 12–54 weeks of resistance training (Kitsuda et al., 2021). In intervention studies, maximum strength of older men and women increased by 7%–226% after undergoing 9–26 weeks of strength training (Frontera et al., 1988; Doherty, 2003). MLEX training over 24 weeks also increased lower body strength (20%), gait speed (19%), and physical activity level (35%) in elderly participants (Ng et al., 2015). For practical application, the prescription of MLEX needs to consider the mode of exercise, which exerts different profile of loading on the MSK system, depending on the nature of movement during execution.

Tai Chi Exercise (TCE) has been adapted for health promotion globally (Zheng et al., 2015; Hong et al., 2000). There are >500 publications on the health benefits of TCE, with 94% showing positive health outcomes, 5% with uncertain effects and <1% with negative effects (Yang et al., 2015). The slow, controlled and multi-directional weight-bearing movements make TCE a suitable and safe form of MLEX for the elderly (Lan et al., 2001; Lan et al., 2013). For example, 3–12 months of TCE improved muscular strength and functions (Lan et al., 2001; Lan et al., 2013; Lan et al., 1998; Lan et al., 2000; Lan et al., 1996; Douglas et al., 2017; Lum and Barbosa, 2019) and decreased the rate of falls and injury-related falls by 40%–50%, compared with sedentary individuals (Lomas-Vega et al., 2017) and stretching exercises alone (Li et al., 2019). Compared with no activities, brisk walking, and traditional dance, 3–8 months of TCE resulted in greater improvement in muscular endurance and strength and BMD in elderly men and women (Song et al., 2014; Zou et al., 2019). This evidence supports the notion that TCE offers a good option for MLEX to promote MSK health for elderly people.

Besides load exposure, physiological adaptations to MLEX are also influenced by exercise volume, which is a function of weekly exercise frequency and duration (min). Elderly participants who performed 12 weeks of multi-component exercise for 150 min/week – 240 min/week showed better quality of life, compared with 100 min/week – 180 min/week of exercise (Rugbeer et al., 2017). Over the same period of 12 weeks, 180 min/week of multi-component exercise had greater benefits than 90 min/week in terms of body weight loss, coordination, cardiorespiratory fitness, body composition, balance and muscular endurance (Nakamura et al., 2007). In a longer term study involving 18 months of multi-component exercise, an exercise volume of >120 min/week showed better improvement than <120 min/week in BMD and LBM of elderly women (Kemmler and von Stengel, 2013). The evidence presented suggests a positive association between exercise volume and a wide range of health benefits, and that the importance of considering both load exposure and exercise volume when prescribing MLEX to promote MSK health and functions in elderly people.

Current evidence on the effects of MLEX on MSK health are derived primarily from multi-component exercises that are prescribed and executed in controlled experimental settings. However, the experimental approach may not accurately reflect the exercise behaviour of elderly people in natural community settings, where participation is usually self-directed, with varied exercise volumes, depending on personal, social, and environmental factors. The effects of community-based exercise participation on MSK health in elderly people are not well-studied. Of particular interest is the comparison between TCE and other forms of MLEX, which is lacking in research evidence. Therefore, the aim of this study was to investigate the effects of exercise mode and volume on MSK health and functions in elderly men and women participating in community-based exercise programs.

## 2 Methodology

### 2.1 Study participants and groups

A cross-sectional design was used to compare body composition, and MSK health and functions in N = 256 elderly men (n = 86) and women (n = 170). All participants were >50 years old and had been sedentary or participating in self-directed or community-based exercise programs for >2 years at the time of this study. Sedentary lifestyle and exercise behavior were determined through a self-declared exercise history. Sedentary participants (SE, n = 60) performed no exercise or <30 min of light to moderate intensity exercise weekly for >2 years. Exercise was defined as physical activity that is planned, structured, and repetitive and aimed at improving physical fitness (Garber et al., 2011).

The exercise participants were recruited mainly from exercise programs/classes that were offered at community exercise facilities in Peoples’ Association Community Clubs and Active SG Facilities. Peoples’ Association offers community exercise classes through 107 community clubs around Singapore and Active SG offers a different variety of community exercise classes and sport facilities through nineteen sport centers around the island. Singapore residents are charged highly subsidized rates for participating in programs offered by these organizations, which are the main service providers for community-based exercise programs in the country. Self-directed participation in brisk walking, jogging, or running exercises were recruited through community network.

#### 2.1.1 Exercise groups

The exercise participants were assigned to the aerobic exercises (AE, n = 62), muscle strengthening exercises (MSE, n = 71), or the Tai Chi exercise (TCE, n = 63) groups, based on their history of exercise participation. AE included participants from self-directed or organized brisk walking, jogging, and running programs, and MSE comprised participants from dance exercises classes (a.k.a. multi-component exercise), self-directed or organized calisthenics and gym exercise classes. TCE participants were recruited from structured group programs offered in the community. All participants had participated in the respective exercise mode on a weekly basis for >2 years. Exercise volume was also derived from the exercise history of the participants, and not pre-determined by the study, to truly reflect the exercise behavior. Recruiting participants from different exercise programs and facilities provides a more realistic reflection on the interaction between exercise and MSK health from the public health perspective. The sum of community exercise participation conducted across the country would influence the interaction between exercise and MSK health in this population.

Participants in all four groups were matched as closely as possible, based on sex and age in 5-year intervals. Participants with common chronic disease (e.g., hypertension, diabetes, and hypercholesterolemia) that were well-controlled with medication were included in the study. However, cigarette and alcohol addictions, diagnosed musculoskeletal diseases (e.g., sarcopenia and osteoporosis) and the use of medication or dietary supplements that could affect body composition and bone health (e.g., steroids, proteins, weigh loss medication) were excluded from the study. All participants were able to perform their daily living activities (e.g., climbing stairs, household chores, and commuting) independently without any physical aide. The procedures of this study were approved by the Institutional Review Board at Nanyang Technological University (IRB-2018-05-019-01), and the participants gave their consent after being briefed on the procedures, risks and benefits of the study, and their rights as research volunteers. The age, sex distribution, anthropometry, and exercise history are shown in Table 1.

**TABLE 1:** Characteristics of participants: Mean (SD) age, height and body mass index, waist-hip ratio, blood pressure, fasted blood glucose and exercise volume.

Parameters	All subjects	Activity groups				Exercise volumes (min/week)			
		MSE	AE	TCE	SE	Min	Low	Mod	High
Sample size	244	66	59	59	60	60	67	59	58
Age (years)	64.6 (5.2)	64.4 (6.3)	63.9 (4.5)	64.7 (4.4)	65.6 (5.1)	65.6 (5.1)	63.9 (5.2)	64.7 (5.3)	64.4 (5.1)
Height (cm)	160.61 (8.13)	161.43 (8.66)	161.26 (7.35)	160.20 (8.23)	159.5 (8.20)	159.46 (8.20)	161.59 (7.58)	160.17 (7.04)	161.10 (9.61)
Body mass index	22.4 (3.1)	22.6 (2.7)	21.9 (3.0)	22.1 (3.5)	23.2 (2.9)	21.2 (2.9)	22.0 (3.1)	22.2 (2.8)	22.4 (3.3)
Waist-Hip ratio	0.8 (0.1)	0.9 (0.1)	0.8 (0.1)	0.9 (0.1)	0.8 (0.1)	0.8 (0.1)	0.8 (0.1)	0.8 (0.1)	0.9 (0.1)
Systolic blood pressure (mmHg)	125 (13)	127 (12)	122 (12)	125 (16)	124 (13)	124 (13)	125 (12)	124 (15)	127 (14)
Diastolic blood pressure (mmHg)	73 (7)	73 (7)	73 (8)	74 (8)	73 (7)	73 (7)	74 (7)	73 (8)	73 (7)
Fasted blood glucose (mmol/L)	5.2 (0.5)	5.3 (0.6)	5.1 (0.3)	5.1 (0.4)	5.1 (0.4)	5.1 (0.4)	5.2 (0.5)	5.1 (0.5)	5.2 (0.5)
Exercise volume (min/week)	155.34 (143.41)	181.67 (112.47)	213.93 (148.57)	225.25 (125.30)	NRPA	NRPA	96.60 (24.08)	181.44 (25.73)	357.33 (121.06)

The main and interaction effects of Group and Exercise volume were not significant in these parameters. “Min” = minimum volume, “Mod” = moderate volume, NRPA, no regular physical activity.

### 2.2 Trial procedures

After giving their informed consent, the participants were given an appointment to attend a laboratory session, which started at about 0830 h and lasted for 3–4 h. The participants were instructed to maintain normal diet and sleep routines for 24 h and to refrain from physical exercise for 48 h before attending the trial. They also refrained from consuming nutritional supplements and caffeine and observed an overnight fast for 24 h before the trial. Upon arrival at the laboratory, the participant declared his/her wellness and were given time to void their bowels and bladders before undergoing the trial procedures. Those who were unwell had their trials rescheduled to another day.

Fasted blood sample (16 mL) was collected with venipuncture at the forearm vein by a phlebotomist, followed by measurement of body weight (BW) in underwear with a weighing scale (Seca Clara 803, Seca Gmbh & Co. KG., Germany). As the trial lasted 3–4 h and the participants came in a fasted state**,** light snacks (buns and sandwiches) and beverages were consumed after blood sampling and body weight measurement, to ensure that they were not energy-depleted to perform the physical function tests. The participants rested for about 30 min after consuming the snacks before proceeding with the trial. At the end of the trial, the participants were observed for 30 min, and declared his/her wellness and had their blood pressure measured before departing the laboratory. Those who were unwell or had abnormal blood pressure were observed further. Where necessary, they were brought to a medical clinic/hospital to receive the appropriate care.

### 2.3 Measurements

#### 2.3.1 Height, blood pressure, body composition and bone mineral density

Height was measured using stadiometer (Seca 213, Seca GmbH & Co. KG., Germany) and brachial blood pressure was measured at the left arm using a pulse wave analysis device (SphymocoCor Xcel, AtCor Medical, Australia), after 5 min rest in supine position. Waist and hip circumferences were measured using a Gulick tape with the participants in underwear and the readings were used to calculate waist-hip ratio (WHR). Body mass index (BMI) was calculated using the ratio of BW (kg) and the square of height (m^2^). Body composition, comprising percentage of body fat (BF%) and lean body mass (LBM%) was measured using the dual energy absorptiometry (DXA) machine (Horizon-W, Hologic Inc., United States), with the participant in supine position and fully clothed. Total bone mineral density (BMD) and BMD at the lumbar spine, neck of femur, and total hip were measured concurrently with the measurement of body composition using the DXA.

#### 2.3.2 Biological sample collection and bioassays

Venous blood (16 mL) was collected from the forearm vein and store in plain (10 mL) and ethylynediamimetetraacetic acid (EDTA, 6 mL) tubes. The blood in plain tubes (4 mL) was left to clot at room temperature and centrifuged at 3,500 rpm for 10 min, under 4°C. The serum supernatant was aliquoted (200 uL) into microtubes for storage in −80°C, and a portion of the stored serum (600 uL) was sent to a licensed pathology laboratory for analysis of calcium concentration (Quest Laboratories Pte Ltd.). Whole blood (53 uL) was also used to measure full blood count (ACT 5 Diff CP, Beckman Coulter, California) and blood glucose (1 drop, ACCU-CHEK Performa Meter, Roche Diagnostics, Switzerland). Blood in the EDTA tube was centrifuged at 3,500 rpm for 10 min, under 4°C. The plasma supernatant was aliquoted (200 uL) into microtubes for storage in −80°C.

Plasma concentrations of type I Collagen Cross-Linked C-Telopeptide (CTX-1) (Serum Crosslaps^®^ CTX-I ELISA, Immunodiagnosticsystems), a biomarker for bone resorption, and bone alkaline phosphatase (BAP, Ostase^®^ BAP EIA, Immunodiagnosticsystems), a biomarker for bone formation, were analysed using ELISA with commercial kits according to manufacturer instructions. Plasma concentration of C-reactive protein (CRP; Invitrogen, ThermoFischer Scientific) was analysed using enzyme-linked immunosorbent assay (ELISA) with commercial kits, according to instructions of the manufacturer.

#### 2.3.3 Physical performance and function tests

##### 2.3.3.1 Hand grip strength

Participants performed the test seated upright while holding a hand dynamometer (Jamar Plus Digital Hand Dynamometer, Patterson Medical Ltd., United States) on the dominant arm, with the elbow flexed at about 90°. The test required the participant to squeeze the handle of the dynamometer with maximum effort. The participants were given to be familiar with procedure and instrument before performing the test 4 times, with 1 min rest between each attempt. The average score, after excluding scores with poor execution, was taken as the result of the test.

##### 2.3.3.2 Isometric strength

Isometric strength was measured on a Jackson strength test device (Jackson Strength Evaluation System, Lafayette Instrument Co., Indiana). The correlation between this form of isometric strength test and 1-repetition maximum strength test using an isokinetic machine was 0.91–0.94. The correlation was lower, at 0.63–0.91, when the isometric strength test was compared against a series of manual labor tasks, such as load lifting and push and pull resistant tasks. The lower correlation is due mainly to differences in movement between this isometric strength test and the manual labor tasks. (Alluisi, 1984; Society, 1988; Human Factors Society, 1992; Jackson, 1999). The participant stood upright on the base plate with feet shoulder width apart, back straight, and knees slightly bent, with both arms holding the lifting bar. The lower end of the lifting-chain was attached to a strain gauge located to the bottom of the base plate, and the upper end of the chain was attached to a lifting bar. The length of the lifting chain was adjusted to be fully extended at the starting position. The participant exerted maximum effort by pulling the lifting bar upwards in an upright row exercise movement. Each participant was given time to familiarize with the test procedure and equipment before attempting the test 4 times, with 1 min rest between each attempt. The average score, after excluding scores with poor execution, was taken as the final score.

##### 2.3.3.3 Trunk flexibility

Trunk flexibility was measured using the sit-and-reach test. The participant sat on the floor with feet (without footwear) shoulder width apart and placed against the surface of the sit-and-reach box (floating zero). When ready, he/she flexed the hip and extended the arms as far as possible without bending the knees. As the arms moved forward, the tip of the longest finger would be pushing the marker that glided along the ruler on the sit-and-reach box. The final position was held for 2 s before the distance traveled by the marker was recorded. The participant was given time to warm-up and up to 4 attempts, with 10 s intervals. The average score, after excluding scores with poor execution, was recognized as the final score.

##### 2.3.3.4 One-leg stand balance test

The attribute of balance was measured using the one-leg stand balance stand**.** Participants stood behind the backrest of a chair with arms by the side. When ready, the non-dominant leg was flexed at the knee joint, and he/she attempted to maintain balance by standing on the dominant leg without holding on to any support. The timing began when the foot was lifted and stopped once the free foot touched the ground or when the hand(s) touched the chair for support. The participant was given time to be familiarized with the test before performing 4 attempts standing on the dominant leg. The average score, after excluding scores with erroneous executions, was taken as the final score.

##### 2.3.3.5 Throwing speed and peak power

This test was performed using a 2 kg power ball (Ballistic Ball, Assess2Perform, Colorado) that had an accelerometer embedded within it. The intraclass correlation for this test system was 0.94–0.98, with a day-to-day variation of 2.2%. Peak velocity measured with the power ball achieved a correlation of 0.85–0.94 when compared with 3-D motion analysis and an optoelectronic system (Roe et al., 2018; Sato et al., 2018; Beckham et al., 2019). When thrown, the ball measured peak power and speed. During the test, the participant sat on a chair and held the ball with both hands in front of the chest. When ready he/she threw the ball forward with maximum effort using a forward chest pass. The participant was given time to warm-up and to be familiar with the procedures before the test began. They had up to 6 attempts for this test and the average of the four highest scores were taken as the results for this test.

## 3 Statistics

All data are presented in mean and standard deviation (SD) and analyzed using the Statistics Package for Social Science Version 26 (IBM SPSS Statistics, United States). A two-factor analysis of variance (ANOVA) was used to analyze the main and interaction effects of group (MSE, AE, TCE and SE) and exercise volume. Exercise volume was categorized into “Minimum” (Min, n = 60 from SE group), “Low” (60–120 min/week, n = 67) “Moderate,” (121–239 min/week, n = 59) and “High” (240 min/week – 720 min/week, n = 58) volumes, according to the tertile distribution of weekly exercise volume recorded across the four groups. In the “High” volume group, majority of participants (n = 52/58) performed 240–500 min/week of exercise. Data for the remaining six participants who performed up to 720 min/week of exercise were included in this group for analysis because there was no impact on the outcomes of the statistical analysis. Data from four subjects who performed 800 min/week to 1,250 min/week of exercise were excluded from analysis because they were beyond the norms of exercise volume for the elderly. Data for another four subjects were excluded because their exercise volumes fell between the criterion for “Low” and “Moderate” categories and two other subjects were excluded because of missing data in exercise history. Pairwise comparisons were analyzed using the Bonferroni *post hoc* test when there was a significant main effect. Normal distribution of the data was determined using the Shapiro Wilks test and data that were not normally distributed were analyzed with the Kruskal Willis non-parametric test. The Dunn-Bonferroni *post hoc* test was used for non-parametric *post hoc* analyses. All the data were normally distributed, except for BF% and LBM% group data, which were analysed using Kruskal Willis test. Statistical significance was defined as *p* < 0.05 for all the analyses.

## 4 Results

### 4.1 General profile and exercise history

There was no significant difference in age, BMI, and exercise volume across the exercise groups (Table 1). The subjects were between 50 and 82 years old, with a mean of 63.9 (4.5) to 65.6 (5.1) years old, and BMI was within normal to borderline obesity for all groups. The MSE group had the lowest mean exercise volume (181.67 (112.47) min/week, 95% C.I, 154.02, 209.32), followed by the AE (213.39 (148.57) min/week, 95% C.I, 175.21, 252.65) and TCE (225.25 (125.30) min/week, 95% C.I, 192.60, 257.91) groups. This trend in exercise volume could reflect the nature and normative routine (class structure and duration) of the respective exercise modalities. The main and interaction effects of group and exercise volume were not significant for all blood parameters, i.e., plasma concentrations of CTX-1, BAP, and CRP, and serum concentration of calcium (Data shown in Supplementary Material).

### 4.2 Analyses within male and female participants

The data for BMD and body composition were also analysed separately within male and female participants because of inherent biological differences in these parameters. Among the female subjects only, the main effect of group was significant for total body BMD (*p* = 0.04) and BMD at the lumbar spine (*p* = 0.004). For male subjects, there was a significant main effect of group for BF% (*p* = 0.04) and LBM% (*p* = 0.006), with exercise groups have lower BF% and higher LBM% than the sedentary group (Data shown in Supplementary Material).

### 4.3 Body composition

Mean BF% differed significantly due to the main effects of group (*p* = 0.03) and exercise volume (*p* = 0.02). The highest mean BF% was recorded in the SE (36.82 (7.48) %, 95% C.I, 34.9, 38.8) group, and BF% was lower but equivocal, differing by about 1%, among the TCE (34.4 (6.01) %, 95% C.I, 32.8, 35.9), AE (33.7 (6.9) %, 95% C.I, 31.87, 35.45) and MSE (33.5 (7.8) %, 95% C.I, 31.6, 35.4) groups. Only the difference in mean BF% between the SE and MSE groups were significant (*p* = 0.05) (Table 2). The order of magnitude for mean BF% corresponded negatively with weekly exercise volume for “Min” (36.8 (7.5) %, 95% C.I, 34.9, 38.8), “Moderate” (34.7 (6.6) %, 95% C.I, 32.9, 36.4), and “High” (34.1 (6.4) %, 95% C.I, 32.4, 35.7) categories, with very similar results in the “Moderate” and “High” volume categories. However, the “Low” volume category had the lowest mean BF% (32.9 (7.7) %, 95% C.I, 31.0, 34.8), which differed significantly from the “Min” volume category (*p* = 0.01).

**TABLE 2:** Mean (SD) of body fat percentage (BF%), lean body mass percentage (LBM%), and bone mineral density (BMD) for total body, lumbar spine, total hip, and neck of femur.

Parameters	All subjects	Activity groups				Exercise volumes (min/week)				ANOVA		
		MSE	AE	TCE	SE	Min	Low	Mod	High	Activity group	Exercise volume	Group X exercise volume
Body fat (%)	34.7 (7.1)	33.5 (7.8)	33.7 (6.9)	34.4 (6.0)	36.8* (7.5)	36.8* (7.5)	32.9 (7.7)	34.7 (6.6)	34.1 (6.4)	*p* = 0.03	*p* = 0.01	N/A
Lean body mass (%)	62.2 (6.9)	63.2 (7.6)	63.1 (6.6)	62.5 (5.8)	60.1 (7.2)	60.1 (7.2)	63.7* (7.5)	62.2 (6.4)	62.8 (6.1)	*p* = 0.03	*p* = 0.02	N/A
Total body BMD (g/cm^2^)	1.02 (0.12)	1.06* (0.13)	1.03 (0.12)	0.99 (0.10)	1.00 (0.11)	1.00 (0.11)	1.04 (0.12)	1.03 (0.11)	1.02 (0.13)	*p* = 0.007	Nil	*p* = 0.02
Lumbar spine BMD (g/cm^2^)	0.90 (0.14)	0.93 (0.16)	0.89 (0.15)	0.86 (0.14)	0.87 (0.12)	0.87 (0.12)	0.89 (0.13)	0.90 (0.14)	0.88 (0.17)	*p* = 0.01	Nil	*p* = 0.02
Total hip BMD (g/cm^2^)	0.83 (0.13)	0.86* (0.14)	0.83 (0.14)	0.79 (0.12)	0.83 (0.10)	0.83 (0.10)	0.83 (0.13)	0.84 (0.14)	0.81 (0.14)	*p* = 0.01	Nil	*p* = 0.006
Neck of femur BMD (g/cm^2^)	0.68 (0.10)	0.70* (0.11)	0.69 (0.12)	0.64 (0.08)	0.67 (0.10)	0.67 (0.10)	0.69 (0.10)	0.68 (0.11)	0.67 (0.11)	*p* = 0.005	Nil	Nil

* = *p* < 0.05 and ** = *p*< 0.01, and *** = *p* < 0.001 for pairwise comparisons in the *post hoc* test. The symbols are marked at the group or exercise volume with the higher score.

The main effects of group (*p* = 0.03) and exercise volume (*p* = 0.02) were significant for mean LBM% (Table 2), but no significant pairwise differences were detected in the *post hoc* analysis. Mean LBM% in the MSE (63.2 (7.6) %, 95% C.I, 61.3, 65.1), AE (63.13 (6.7) %, 95% C.I, 61.4, 64.9) and TCE (62.5 (5.8) %, 95% C.I, 60.9, 64.0) groups were very similar, differing by < 1%, but mean LBM% in the SE group (60.1 (7.2) %, 95% C.I, 58.2, 62.0) was distinctly lower by 2.4%–3.1% than the exercise groups. A similar observation between the exercise and sedentary groups was found for exercise volume. Mean LBM% differed by 1%–1.5% between “Low” (63.7 (7.5) %, 95% C.I, 61.9, 65.5), “Moderate” (62.2 (6.4) %, 95% C.I, 60.2, 63.9) and “High” (62.8 (6.1) %, 95% C.I, 61.2, 64.4) exercise volumes, but was lower by 2%–3.6% in the “Min” (60.1 (7.2) %, 95% C.I, 58.2, 62.0) than the higher exercise volume categories.

### 4.3 Bone mineral density

The main effect of group and the interaction effect of group and exercise volume were significant for total body BMD (*p* = 0.007 for main effect and *p* = 0.02 for interaction effects), and BMD at the lumbar spine (*p* = 0.01 for main effect and *p* = 0.02 for interaction effects) and total hip (*p* = 0.01 for main effect and *p* = 0.006 for interaction effects) (Table 2). BMD at the neck of femur also differed significantly between the groups (*p* = 0.005). MSE and AE had consistently higher BMD than TCE and SE in all these anatomical sites, but only the difference in BMD between the MSE and TCE groups was significant in the Hip (*p* = 0.03) and at the neck of femur (*p* = 0.008). There was no clear trend between BMD and exercise volume in these anatomical sites.

### 4.4 Trunk flexibility

**FIGURE 1:**
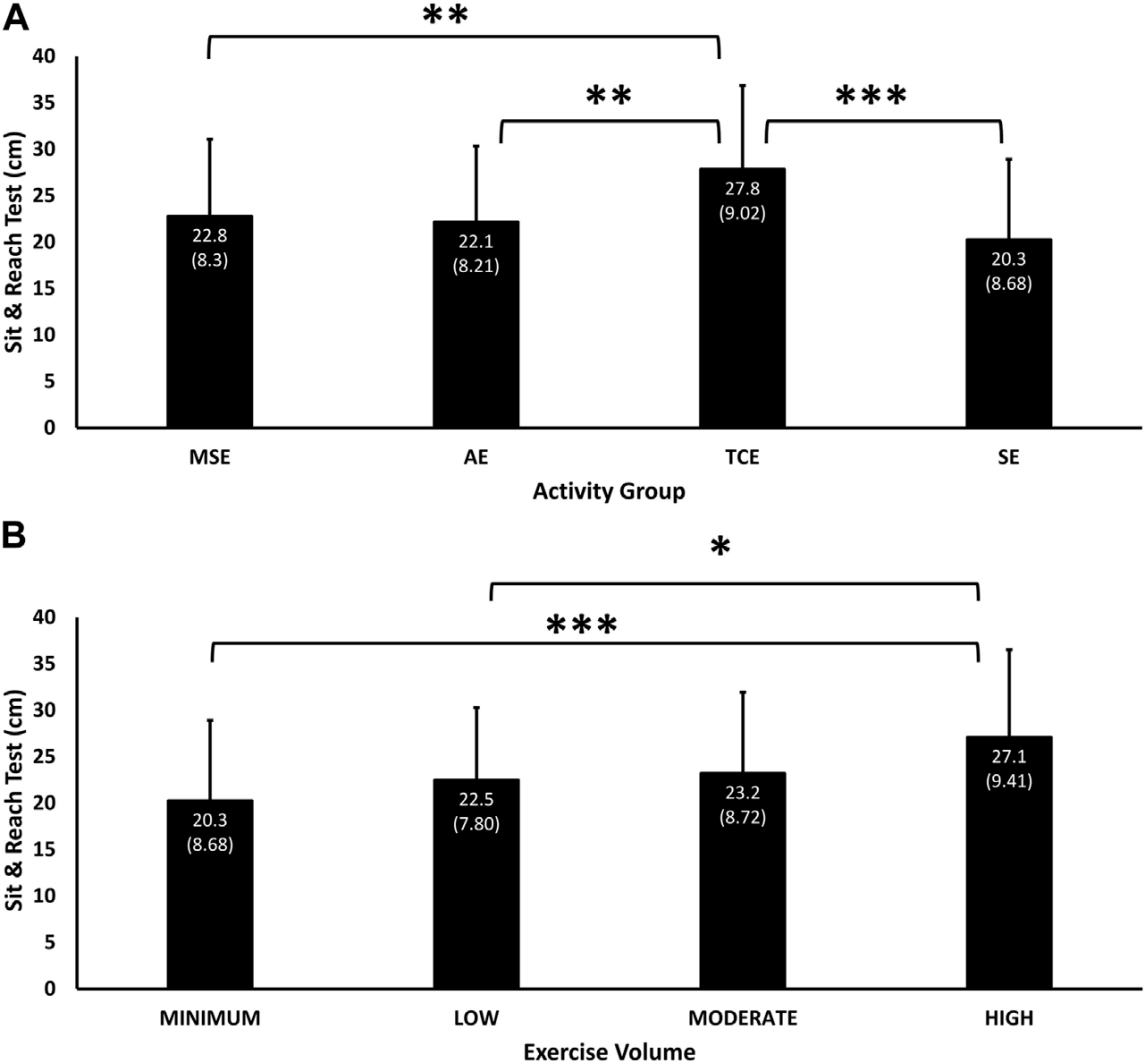
Mean (SD) of performance in the sit-and-reach test according to experimental groups **(A)** and exercise volumes **(B)**. Significant main effect of Group (*p* = 0.003) and Exercise Volume (*p* = 0.04). * = *p* < 0.05 and ** = *p*< 0.01, and *** = *p* < 0.001 significant difference in pairwise comparisons between TCE and the MSE (*p* = 0.007), AE (*p* = 0.002) and SE (*p* < 0.001) groups, and between “High” and “Low” (*p* = 0.018) and “Min” (*p* < 0.001) exercise volumes.

Performance in the sit-and-reach test differed significantly due to the main effects of group (*p* = 0.003) and exercise volume (*p* = 0.04). The TCE group (27.8 cm, 95% C.I, 25.5, 30.2) had the greatest trunk flexibility, followed by MSE (22.8 (8.3) cm, 95% C.I, 20.7, 24.8), AE (22.1 (8.2) cm, 95% C.I, 20.0, 24.3), and SE (20.3 (8.7) cm, 95% C.I, 17.9, 22.6) groups (Figure 1A). The TCE participants had significantly greater trunk flexibility than MSE (*p* = 0.007), AE (*p* = 0.002) and SE (*p* < 0.001) groups. Sit-and-reach test performance corresponded in a dose-response manner with exercise volume (*p* = 0.043), with “High” volume having significantly greater trunk flexibility than “Low” (*p* = 0.018) and “Min” (*p* < 0.001) volumes (Figure 1B)

### 4.5 One-leg stand balance test

**FIGURE 2:**
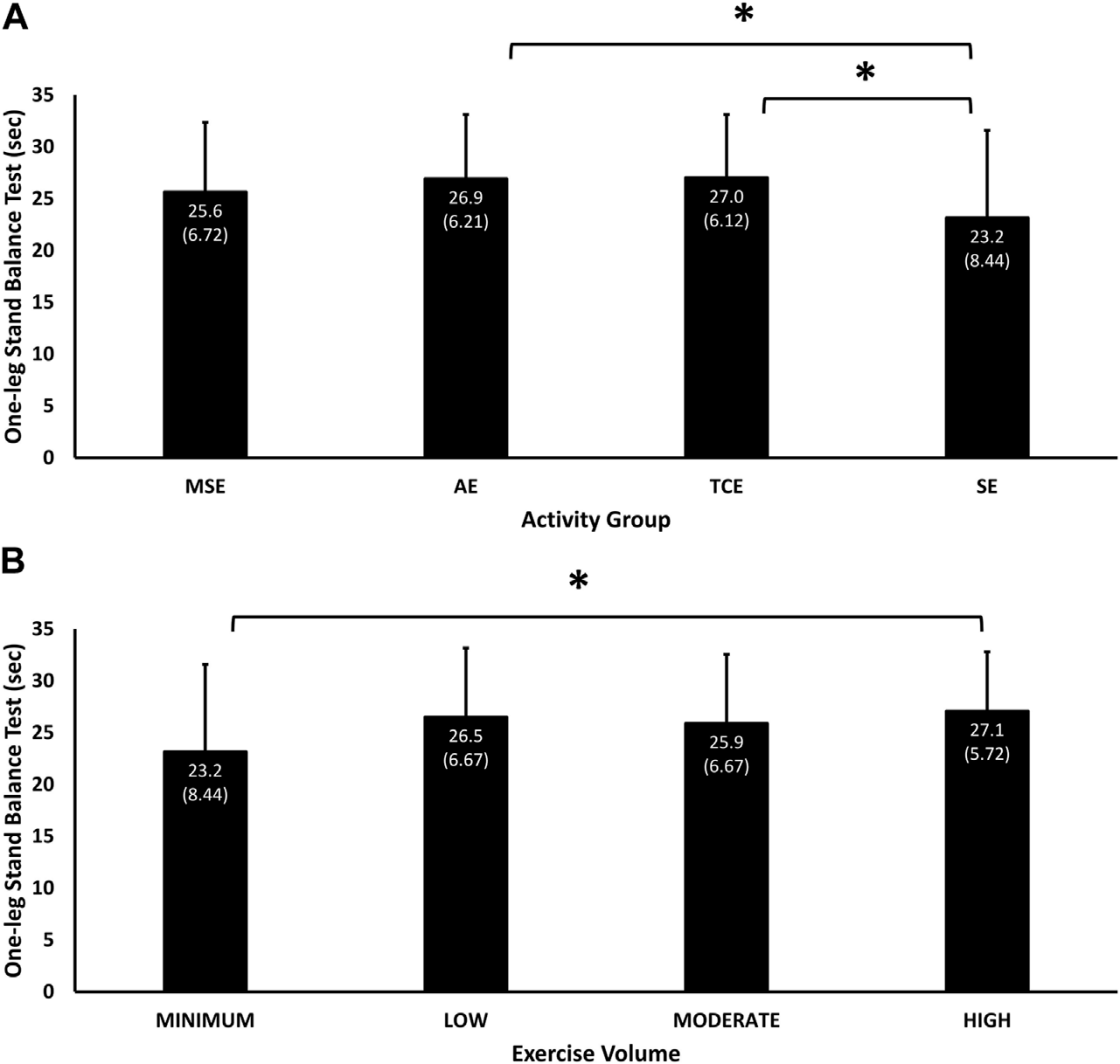
Mean (SD) performance in the one-leg Stand balance test according to experimental groups **(A)** and exercise volume **(B)**. Significant main effect of Group (*p* = 0.01) and Exercise Volume (*p* = 0.03). * = *p* < 0.05 significant difference in pairwise comparisons between TCE (*p* = 0.03) and AE (*p* = 0.03) with the SE group, and between “High” and “Minimum” exercise volumes (*p* = 0.05).

The main effects of group (*p* = 0.01) and exercise volume (*p* = 0.03) were significant for the balance test. The best performance was in the TCE group (27.01 (6.1) sec, 95% C.I, 25.41, 28.60), followed by the AE (26.9 (6.2) sec, 95% C.I, 25.30, 28.54), MSE (25.6 (6.7) sec, 95% C.I, 23.99, 27.29) and SE (23.2 (8.4) sec, 95% C.I, 20.93, 25.37) groups (Figure 2A). Both the TCE (*p* = 0.03) and AE (*p* = 0.03) groups had significantly better balance skills than the SE group. Balance performance also corresponded with exercise volume for the “High” (27.08 (5.72) sec, 95% C.I, 25.58, 28.58), “Moderate” (25.9 (6.7) sec, 95% C.I, 24.15, 27.63) and “Min” (23.2 (8.4) sec, 95% C.I, 20.93, 25.37) categories, with no advantage of “Moderate” over “Low” (26.5 (6.7) sec, 95% C.I, 24.88 28.13) exercise volumes (Figure 2B). The performance of the “High” volume category was significantly better than the “Min” category (23.2 (8.4) sec, *p* = 0.05).

### 4.6 Isometric strength

**FIGURE 3:**
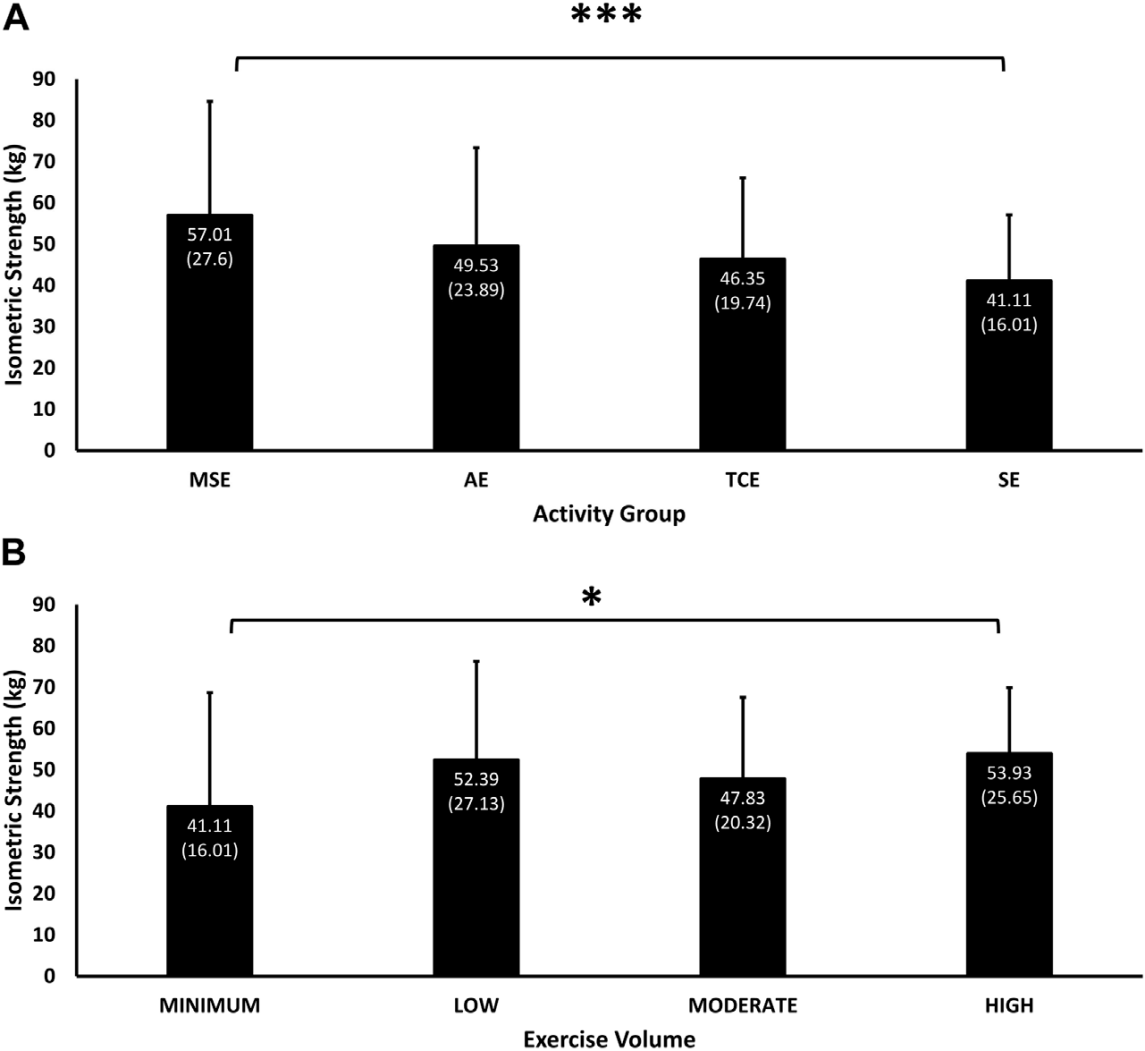
Mean (SD) of performance in isometric strength according to experimental groups **(A)** and exercise volumes **(B)**. Significant main effect of Group (*p* = 0.01) and interaction effect of Group and Exercise Volume (*p* < 0.001). * = *p* < 0.05, and *** = *p* < 0.001 significant difference in pairwise comparisons between MSE and SE group (*p* = 0.001), and between the “High” than the “Low” exercise volumes (*p* = 0.019).

The main effect of group (*p* = 0.01) and its interaction with exercise volume (*p* < 0.001) were significant for the Jackson isometric strength test, which was highest among MSE participants (57.01 (27.60) kg, 95% C.I, 50.22, 63.79), followed by AE (49.64 (24.46) kg, 95% C.I, 43.21, 56.08), TCE (46.76 (19.92) kg, 95% C.I, 41.48, 52.05) and SE (41.11 (16.01) kg, 95% C.I, 36.89, 45.32) participants (Figure 3A). Isometric strength was significantly higher in the MSE than in SE group (*p* = 0.001). Isometric strength corresponded with exercise volume in the “High” (53.93 (25.65) kg, 95% C.I, 47.19, 60.68), “Moderate” (47.83 (20.32) kg, 95% C.I, 42.48, 53.17) and “Min” (41.11 (16.01) kg, 95% C.I, 36.89, 45.32) categories. However, isometric strength was higher (not significant) with “Low” (52.39 (27.13) kg, 95% C.I, 45.67, 59.11) than with “Moderate” exercise volumes (Figure 3B) and was significantly higher in the “High” than the “Min” exercise volume categories (*p* = 0.019).

### 4.7 Handgrip strength

**FIGURE 4:**
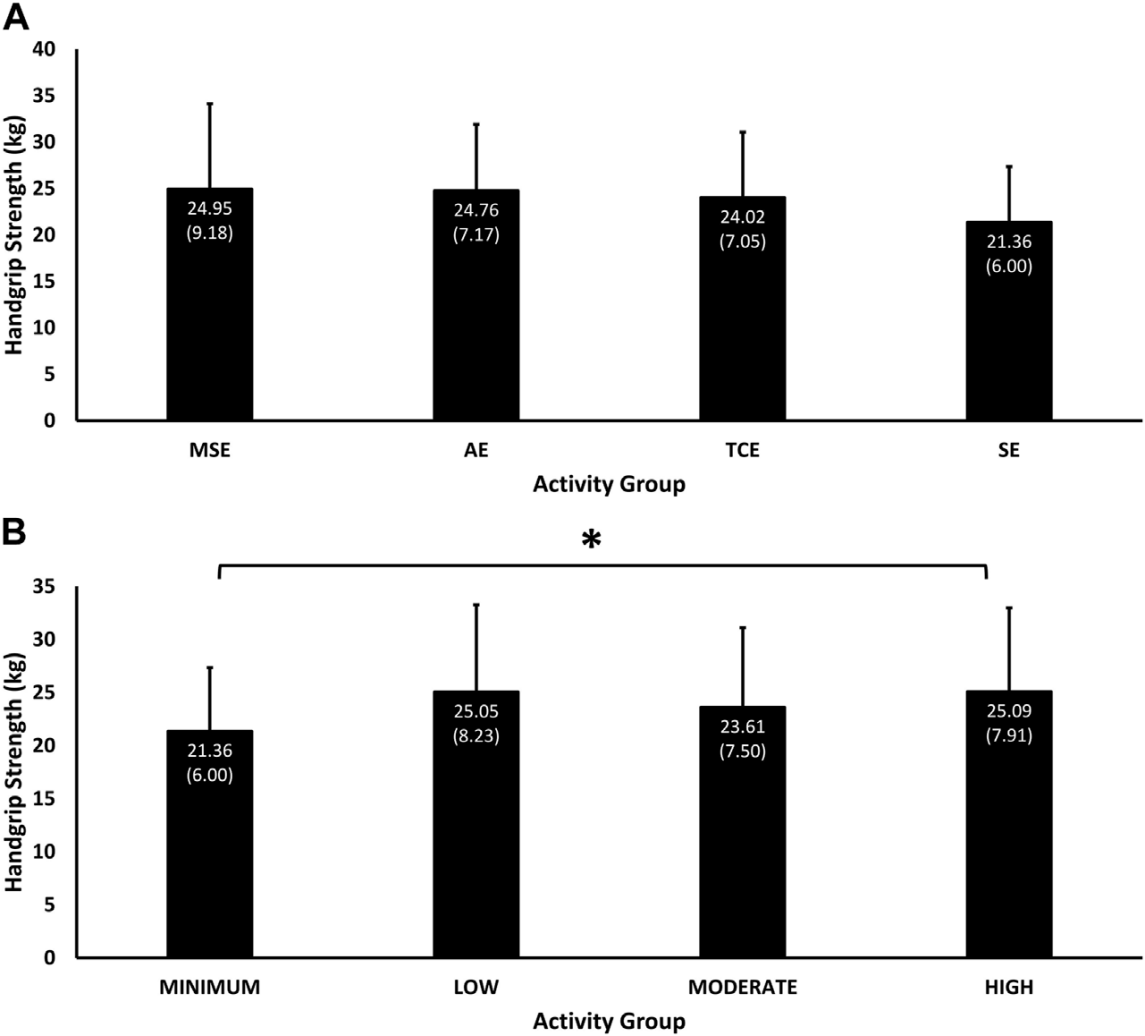
Mean (SD) of handgrip strength performance according to experimental groups **(A)** and exercise volumes **(B)**. Significant interaction effects of Group and Exercise Volume (*p* = 0.03). * = *p* < 0.05 significant difference in pairwise comparison between “Minimum” and “High” exercise volumes (*p* = 0.04).

Handgrip strength differed significantly due to the interaction effect of group and exercise volume (*p* = 0.03), and was highest in MSE group (24.95 (9.18) kg, 95% C.I, 22.68, 27.23), followed by the AE (24.76 (7.17) kg, 95% C.I, 22.87, 26.64), TCE (24.02 (7.05) kg, 95% C.I, 22.16, 25.87), and SE (21.36 (6.00) kg, 95% C.I, 19.78, 22.94) groups (Figure 4A). Handgrip strength was positively associated with exercise volume in the “High” (25.09 (7.91) kg, 95% C.I, 22.97, 27.21), “Moderate” (23.61 (7.50) kg, 95% C.I, 21.66, 25.56) and “Min” (21.36 (6.00) kg, 95% C.I, 19.78, 22.94) categories, but handgrip strength in the “Low” exercise volume category (25.05 (8.23) kg, 95% C.I, 23.02, 27.07) performed better than the “Moderate” category (Figure 4B). Only the difference in handgrip strength between the “Min” and “High” exercise volume categories was significant (*p* = 0.04).

### 4.8 Peak throwing speed

**FIGURE 5:**
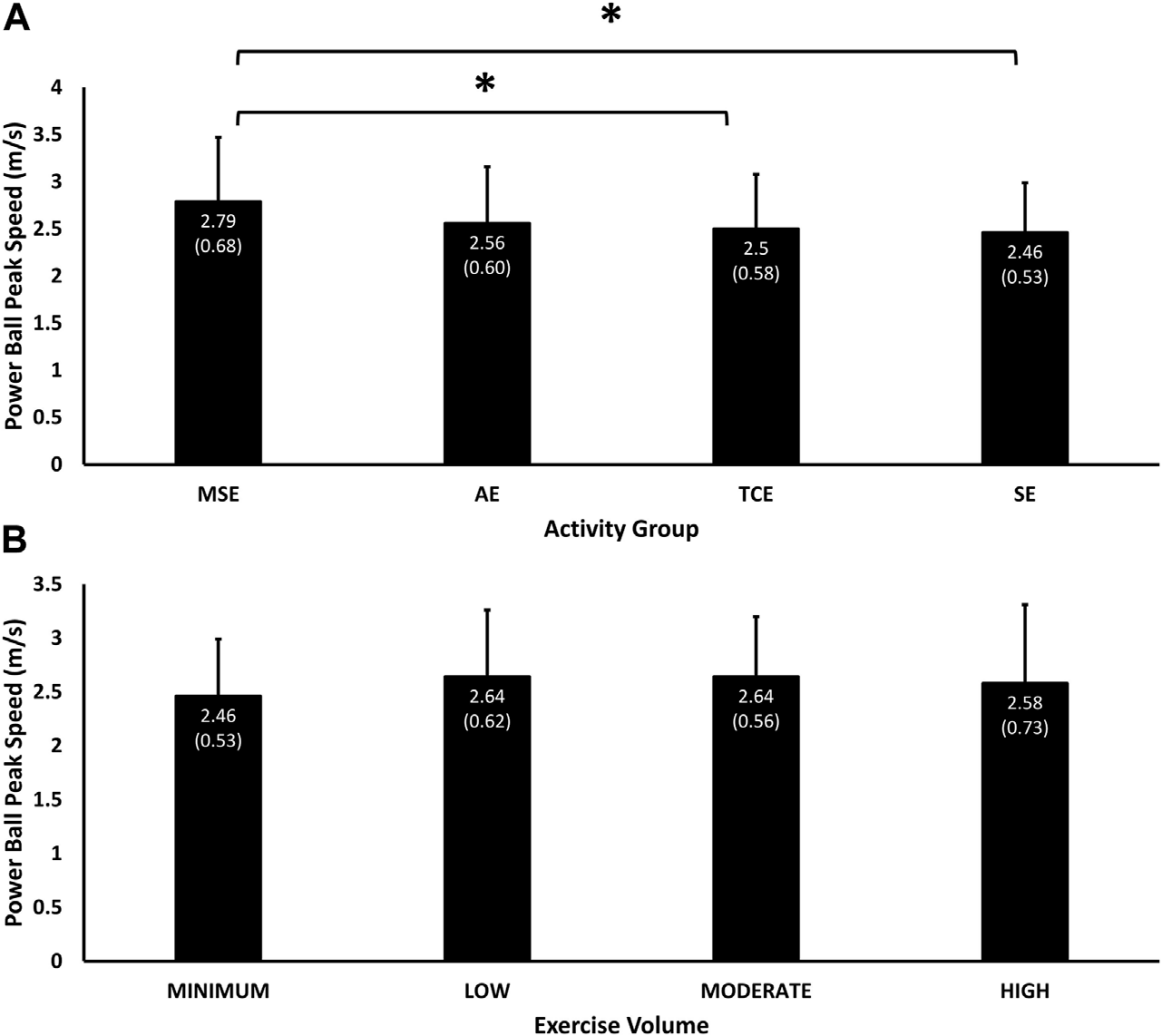
Mean (SD) of performance in throwing speed according to experimental groups **(A)** and exercise volumes **(B)**. Significant main effect of Group (*p* = 0.005) and interaction effect of Group and Exercise Volume (*p* = 0.003). * = *p* < 0.05 significant difference in pairwise comparisons between MSE and the TCE (*p* = 0.05) and SE (*p* = 0.02) groups.

Peak throwing speed with a 2 kg ball was influenced significantly by the main effect of group (*p* = 0.005) and the interaction effect of group and exercise volume (*p* = 0.003). Throwing speed was the highest among MSE participants (2.79 (0.68) m/sec, 95% C.I, 2.62, 2.96), followed by AE (2.56 (0.60) m/sec, 95% C.I, 2.39, 2.73), TCE (2.50 (0.58) m/sec, 95% C.I, 2.34, 2.65) and SE (2.46 (0.53) m/sec, 95% C.I, 2.32, 2.61) participants (Figure 5A). Throwing speed in the MSE group was significantly higher than the TCE (*p* = 0.05) and SE (*p* = 0.02) groups. Throwing speed corresponded positively with exercise volume in the “Moderate” (2.64 (0.56) m/sec, 95% C.I, 2.49, 2.79), “Low” (2.64 (0.62) m/sec, 95% C.I, 2.48, 2.80), and “Min” (2.46 (0.53) m/sec, 95% C.I, 2.32, 2.61) categories, but not in the “High” volume category (2.58 (0.73) m/sec, 95% C.I, 2.38, 2.78), which was only faster than the “Min” volume category (Figure 5B).

### 4.9 Peak power

**FIGURE 6:**
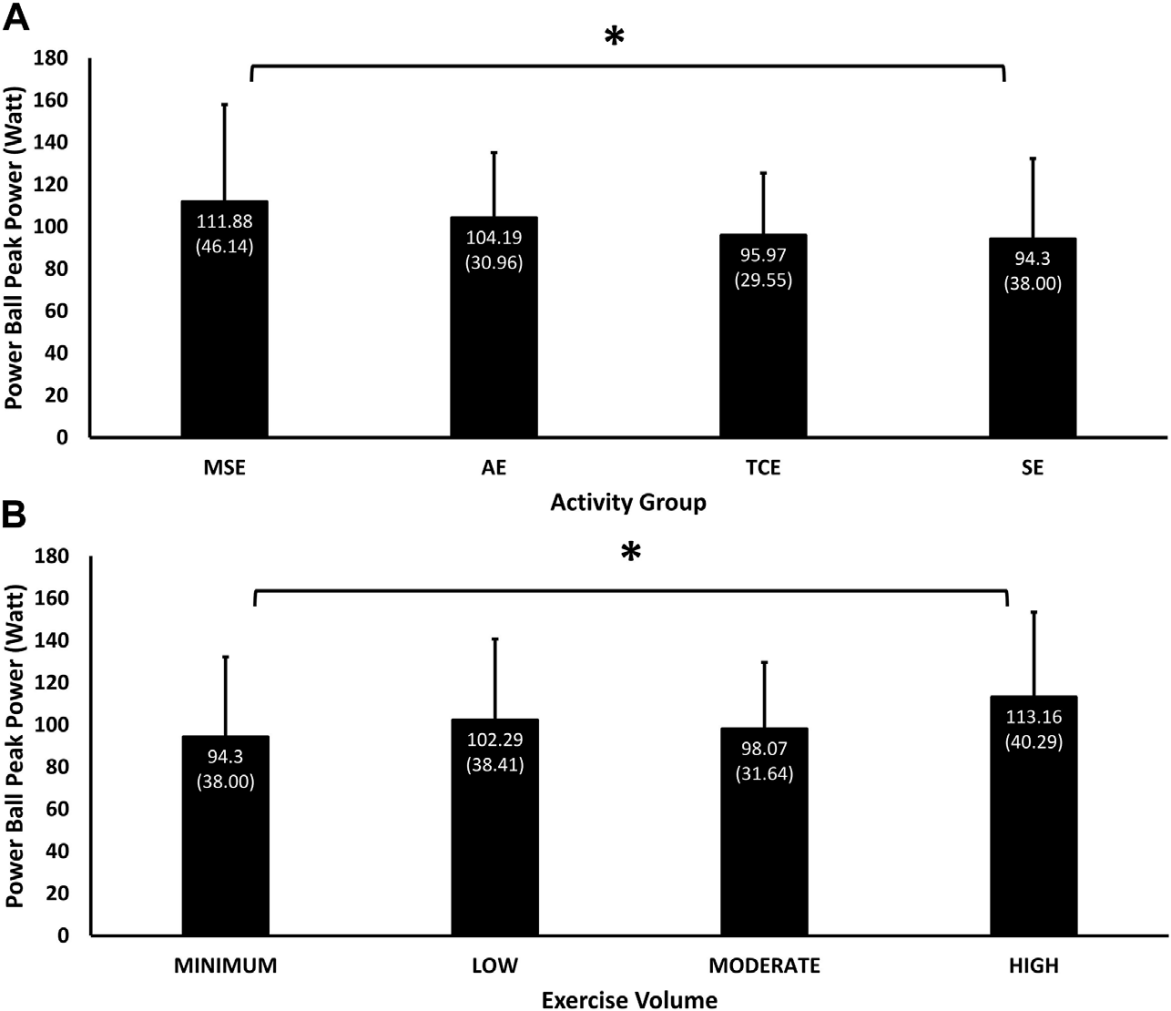
Mean (SD) of performance in throwing peak power according to experimental groups **(A)** and exercise volumes **(B)**. Significant main effects Group (*p* = 0.01) and Exercise Volume (*p* = 0.02), their interaction (*p* = 0.001) * = *p* < 0.05 significant difference in pairwise comparisons between the MSE and SE groups (*p* = 0.05), and between “Minimum” and “High” exercise volumes (*p* = 0.04).

Peak power measured during the 2 kg ball throw was influenced significantly by the main effects of group (*p* = 0.01) and exercise volume (*p* = 0.02) and their interaction (*p* = 0.001). MSE participants (111.88 (46.14) W, 95% C.I, 99.96, 123.80) had the highest peak power, followed by AE (104.19 (30.96) W, 95% C.I, 95.48, 112.90), TCE (95.97 (29.55) W, 95% C.I, 87.98, 103.96), and SE (94.30 (38.00) W, 95% C.I, 84.30, 104.29) participants (Figure 6A), but only the difference between the MSE and SE groups was significant (*p* = 0.05). Peak power was positively associated with exercise volume in the “High” (113.16 (40.29) W, 95% C.I, 101.94, 124.37), Moderate” (98.07 (31.64) W, 95% C.I, 89.68, 106.47) and “Min” (94.30 (38.00) W, 95% C.I, 84.30, 104.29) categories, but participants with “Low” volume (102.29 (38.41) W, 95% C.I, 92.10, 112.48) performed better than those with “Moderate” volume” (Figure 6B). Only the difference in peak power between “Min” and “High” volumes was significant (*p* = 0.04).

## 5 Discussion

Weekly participation in community-based MLEX programs for >2 years was associated with lower BF% and higher LBM% than sedentary lifestyle, with no distinct advantage between the exercise groups. These results add to current evidence showing the beneficial effects of MLEX for promoting body composition health (Frontera et al., 1988; Staron et al., 1991; Lan et al., 1996; Harridge et al., 1999; Thompson et al., 2004; Lee et al., 2012). BF% was higher by 2.5%–3.3% in the sedentary group, which translates to 1.5–2 kg more fat mass in sedentary, than in exercise participants. Similarly, mean LBM% in the SE group was lower than the exercise groups by 2.4%–3%, which translates to 1.4–1.75 kg lesser LBM. These differences in fat and lean masses may have significant implications on health risks that are associated with obesity and sarcopeniay in elderly people. Our results suggest that these health risks can be mitigated by participating in a variety of community-based MLEX modalities that are equivalent to those in this study.

All three categories of exercise volume were associated with lower BF% and higher LBM% than sedentary lifestyle. However, among the exercise groups, “High” exercise volume had no advantage over “Moderate” volume for BF%, and “Low” exercise volume had the greatest benefit for BF% and LBM%. Another study also reported greater increase in LBM with 120 min/week, compared with <120 min/week of multi-component exercise for 18 months, and LBM in both exercise volumes was significantly higher than sedentary subjects (Kemmler and von Stengel, 2013). The “Low” exercise volume participants in the current study were dominated by the MSE group, which had the lowest for BF% and highest for LBM%. These results are likely due to the specificity of muscle strengthening exercises in promoting gains in LBM. Taken together, these results suggest that there is a positive association between exercise volume and body composition and that “Moderate” exercise volume of >120 min/week of MLEX may be adequate for promoting body composition health in older individuals. For those participating in MSE modes, a lower exercise volume of >60 min/week may be adequate to improve body composition health.

The association between MLEX, BMD, and biomarkers for bone remodelling remains unclear as these results were equivocal between the exercise and sedentary groups and across the categories of exercise volume. These findings are in agreement with current evidence showing no distinct advantage of exercise modalities in inducing the response of biomarkers for bone remodelling, irrespective of the magnitude and volume of impact and loading characteristics on bones (Dolan et al., 2020). However, the lack of advantage with MLEX and exercise volume on BMD contradicted the consensus on the osteogenic effects of load-bearing exercise and exercise volume on bone remodelling (Chodzko-Zajko et al., 2009; Agostinete et al., 2020; Prins et al., 2020; Abilgaard et al., 2022; Beck, 2022). It is possible that a higher dose of exercise volume and load is needed to elicit a higher BMD response in the skeletal system. For example, only women who exceeded the exercise guidelines for Americans by 2–4 times (15–30 MET-h/week) and men who exceeded the guidelines by > 4 four-fold (>30 MET/h/week) had higher BMD at the lumbar spine and proximal femur than sedentary subjects (Whitefield et al., 2015). It is also important to consider other age-related physiological changes that may impede the response of bone remodelling to MLEX training. For example, the effects of menopause and andropause, which are associated with bone degeneration in the elderly population (Woolf and Pfleger, 2003; Ahlborg et al., 2010; Goh and Hart, 2016; Rinonapoli et al., 2021; Vilaca et al., 2022). Therefore, the lack of an association between MLEX and exercise volume with BMD should be read in the context of elderly health and should not suggest that MLEX does not contribute to bone health in all populations. There is abundant evidence supporting the recommendation of MLEX for promoting musculoskeletal health in younger populations (Haskell et al., 2007; Garber et al., 2011; Bull et al., 2020).

Participating in community-based TCE programs for >2 years had a distinct advantage over the other exercise modes and sedentary lifestyle in trunk flexibility. TCE and AE were also associated with better performance in the balance test than sedentary lifestyle, and there are additional benefits in trunk flexibility (TCE) and balance (TCE and AE) with “High” volume of MLEX.

Our results add to the current consensus on the beneficial effects of TCE in promoting flexibility (Hong et al., 2000; Lan et al., 1996; Wehner et al., 2021; Lee et al., 2008; Zou et al., 2017) and balance (Hong, Li, Robinson; Song et al., 2014; Wehner et al., 2021; Zhou et al., 2019; Chewning et al., 2019) in older people (Hong, Li, Robinson). Elderly women who practiced TCE for 12 months had lower incidence rate ratio (IRR) for moderate (IRR = 0.51) and serious injurious falls (IRR = 0.25), compared with stretching exercises over the same period (Li et al., 2019). They also had significantly lower incidence of serious injurious falls (IRR = 0.025) than participants who underwent 12 months of multi-component exercise (Li et al., 2019). These results demonstrated that TCE offers a good choice of exercise for fall prevention and for lowering the risks of acute MSK injuries (Batıbay et al., 2021; Kozlenia and Domaradzki, 2021). Trunk flexibility performance predicted musculoskeletal injury with a 41% accuracy, and every 1 cm decrease in trunk flexibility was associated with a 6% increase in the risk of these injuries (Kozlenia and Domaradzki, 2021). Our results support the promotion of TCE among elderly in the community and to accumulate >240 min/week of TCE to benefit fully from the exercise. The benefit of AE for the balance test has not been reported before and implies that there may be common neural pathways between the balance task and the walking/running movement. This possibly occurs during the momentary unipedal movement in the transfer phase of the walking/running motion. If this reasoning is valid, AE may also offer another mode of exercise for promoting balance and fall prevention for elderly people.

MSE had a distinct advantage in isometric strength and throwing speed and peak power, followed by AE and TCE, and all exercise groups performed better in these muscle functions than SE. The only exception was handgrip strength, where the performance was equivocal (<1%) among the exercise groups, but was 14% higher than the SE group. These results agree with current evidence on the benefits of MSE, AE and TCE in promoting these muscular health and functions (Lavin et al., 2019; Frontera et al., 1988; Lan, Lai, Chen, wong; Lum and Barbosa, 2019; Zou et al., 2019; Wehner et al., 2021; Holviala et al., 2012). Our results also suggest that 60 min/week of MSE or >120 min/week of all three forms of MLEX is sufficient to promote muscular strength, speed and power. When combined with the positive results of MLEX on LBM%, the positive effects of MLEX on muscle performance have important implications for mitigating the risk and progression of sarcopenia. This is because the diagnosis of sarcopenia are premised on muscle quantity (LBM) and quality (muscle functions) (Cruz-Jentof et al., 2010; Bijlsma et al., 2013; Dent et al., 2017; Cruz-Jentoft et al., 2018), which are associated with LBM% and the muscle functions reported in this study. These beneficial effects of MLEX are likely to be observed with >2 years of exercise participation and with an exercise volume of >120 min/week for most forms of MLEX and for 60 min/week for MSE modalities. Clinicians and public health administrators may use this evidence to support the use of MLEX to mitigate the risks and progress of sarcopenia in community settings.

The results in this study need to be interpreted with a few limitations in mind. For example, the use of exercise history to determine mode and volume of exercise participation over ≥2 years is subjected to errors in memory and inconsistency between the participants. This limitation was moderated by recruiting participants primarily from structured exercise classes offered by the two primary service providers in Singapore. These weekly classes would have provided a mental framework to recall exercise history with a higher degree of consistency among the participants. The cross-sectional study design also has a higher degree of inconsistency in the intensity and duration of exercise within and between each group. Moreover, this study investigated the “primary” form of weekly exercise and not the “total” weekly exercise participation, leaving the possibility that participation in other forms of exercise may not be captured in this present data. This error is likely to be small as majority of the exercise participants reported participating only in one form of exercise on a regular basis.

## 6 Conclusion

The present study supports the recommendation for older people to participate in >120 min/week of community-based MSE, AE and TCE programs to protect MSK health against the degenerating effects of ageing. The possibility of achieving the same health benefits with a lower exercise volume (>60 min/week) of MSE deserves further investigation. Participating in >120 min/week of TCE and AE have significant advantage in improving trunk flexibility (TCE) and balance (AE) to lower the risks of falling and musculoskeletal injuries. However, there was no strong association between these MLEX modalities and BMD. This study supports the greater use of community-based MLEX programs to prevent and mitigate the progression of common age-related chronic, such as sarcopenia, obesity, and metabolic disease. Healthcare delivery can now include community exercise resources for the promotion and management of body composition and muscular health.

## Data Availability

The original contributions presented in the study are included in the article/Supplementary Material. Further inquiries can be directed to the corresponding author.
