# Supplementary figures and images for: The effects of community-based exercise modalities and volume on musculoskeletal health and functions in elderly people
Source: Front Physiol. 2023 Jul 10;14:1227502. doi: 10.3389/fphys.2023.1227502 (PMC10363600; doi:10.3389/fphys.2023.1227502)

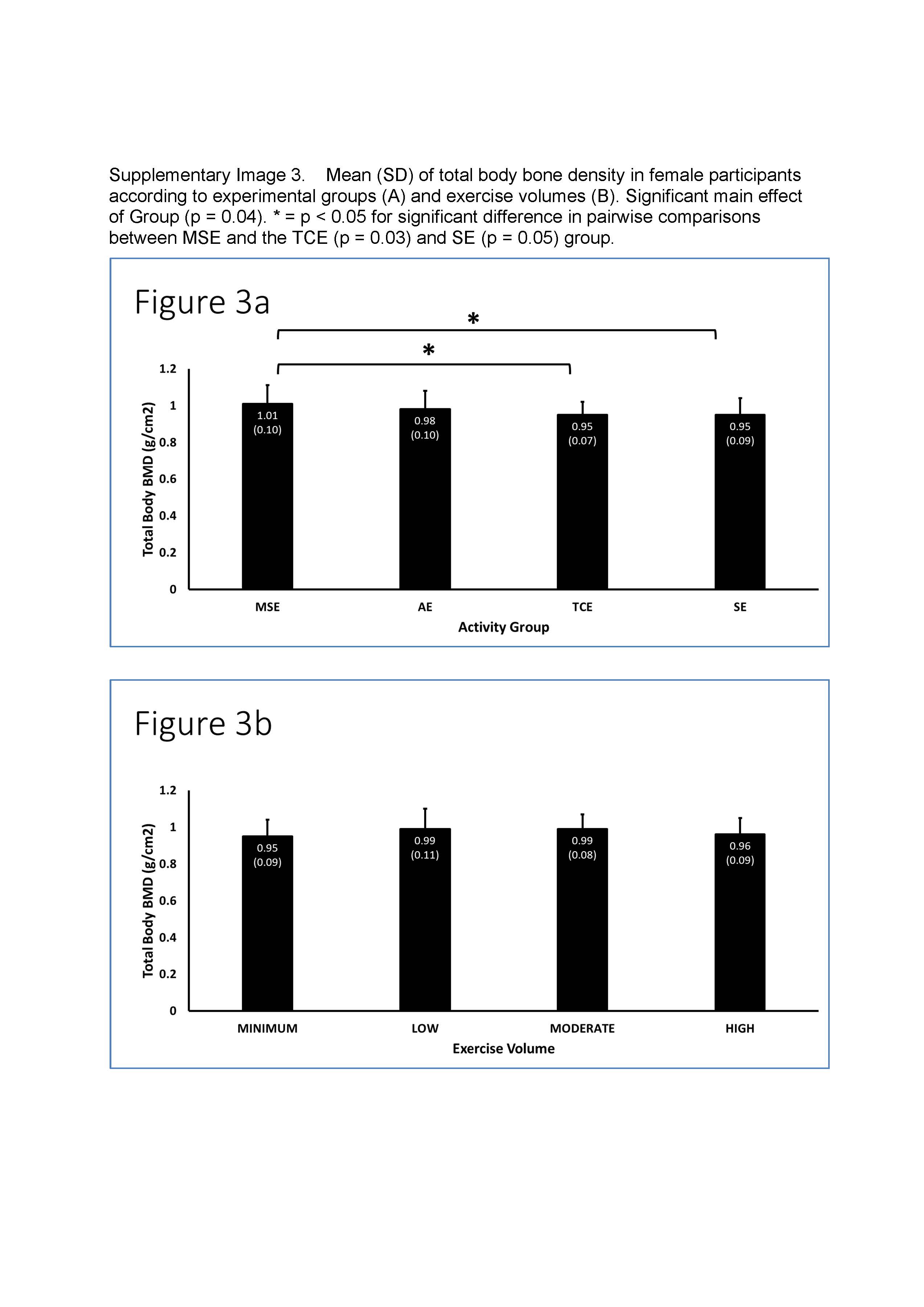

Supplement: Supplementary file 1 [file Figure3.jpg]

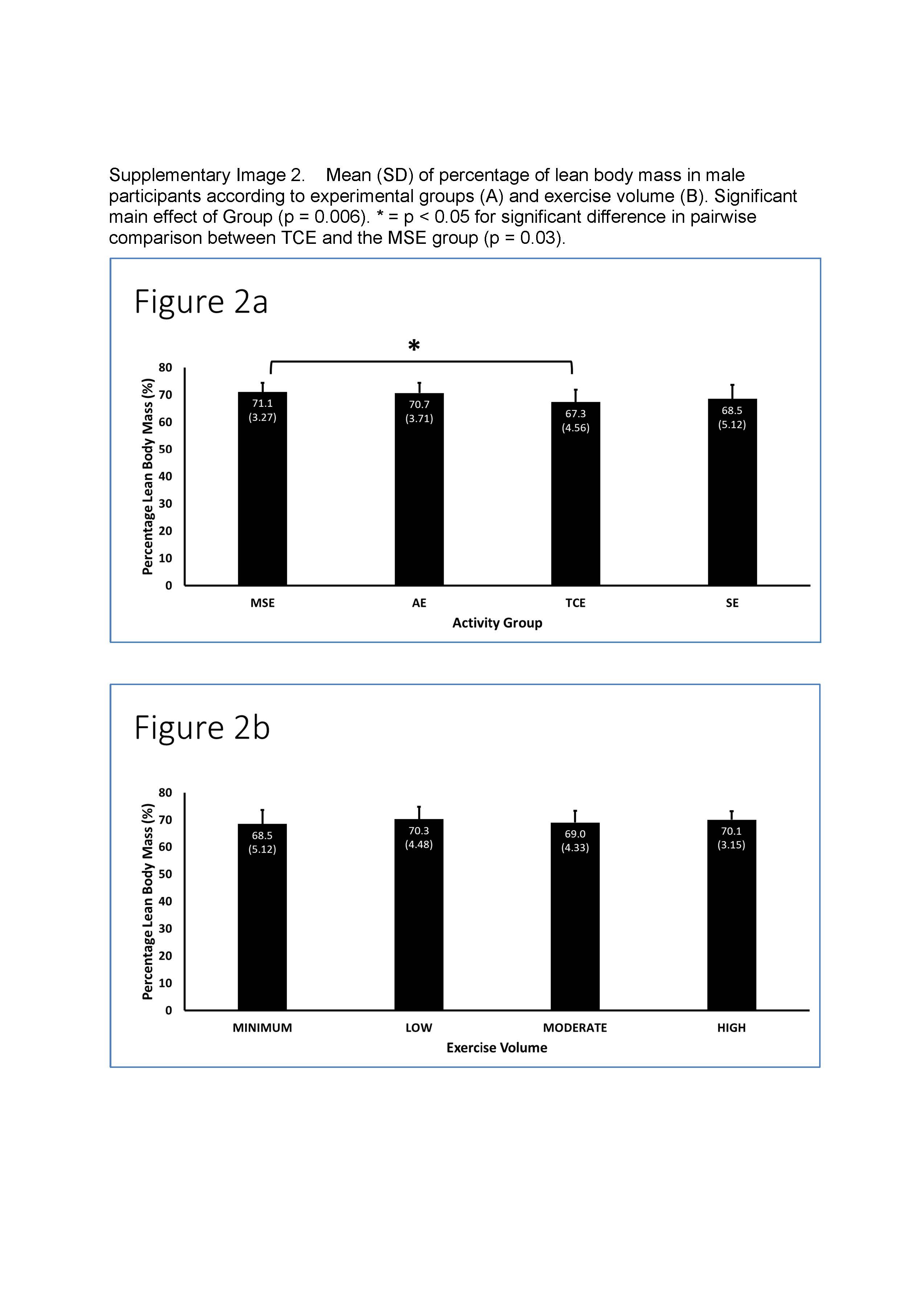

Supplement: Supplementary file 2 [file Figure2.jpg]

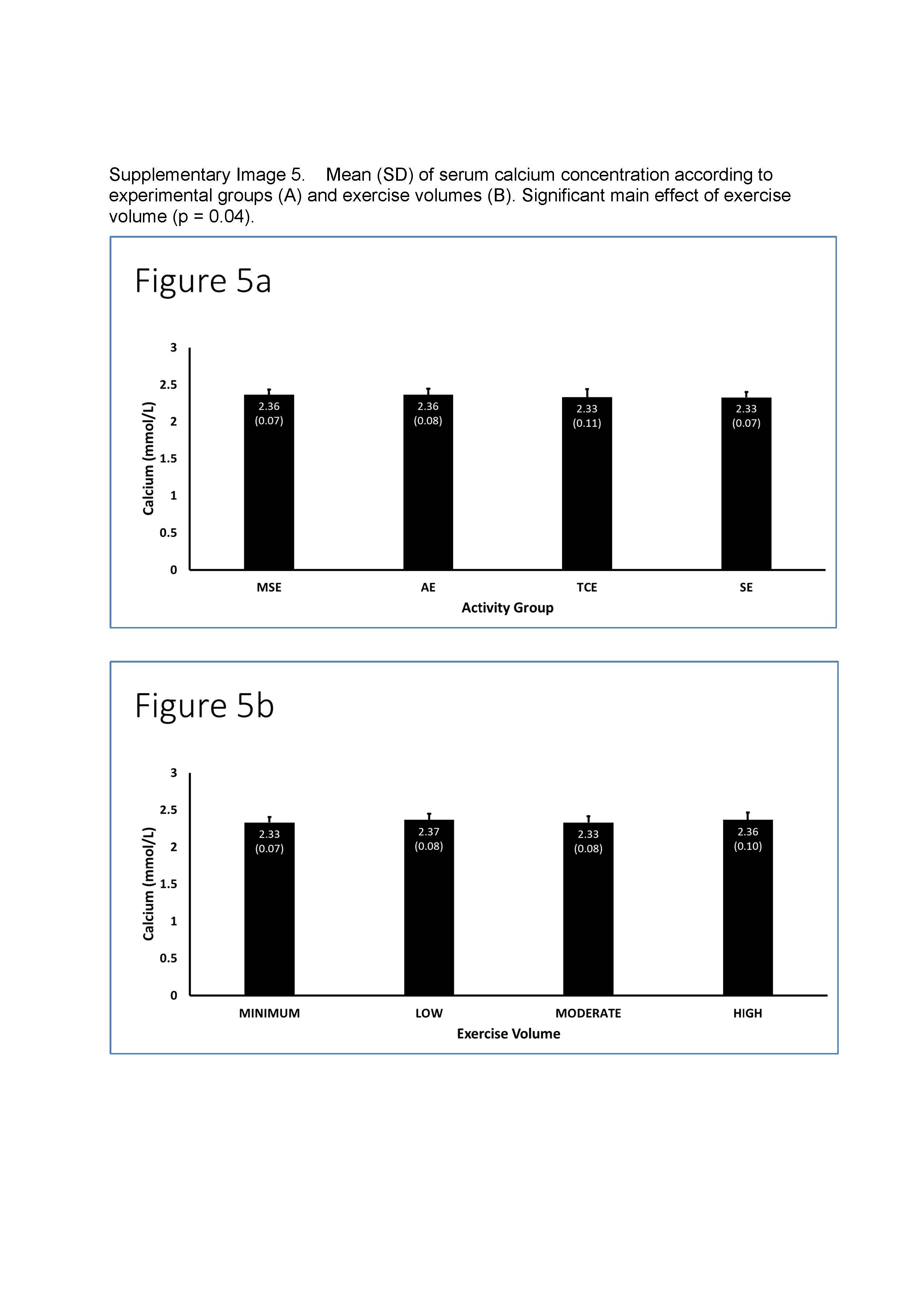

Supplement: Supplementary file 3 [file Figure5.jpg]

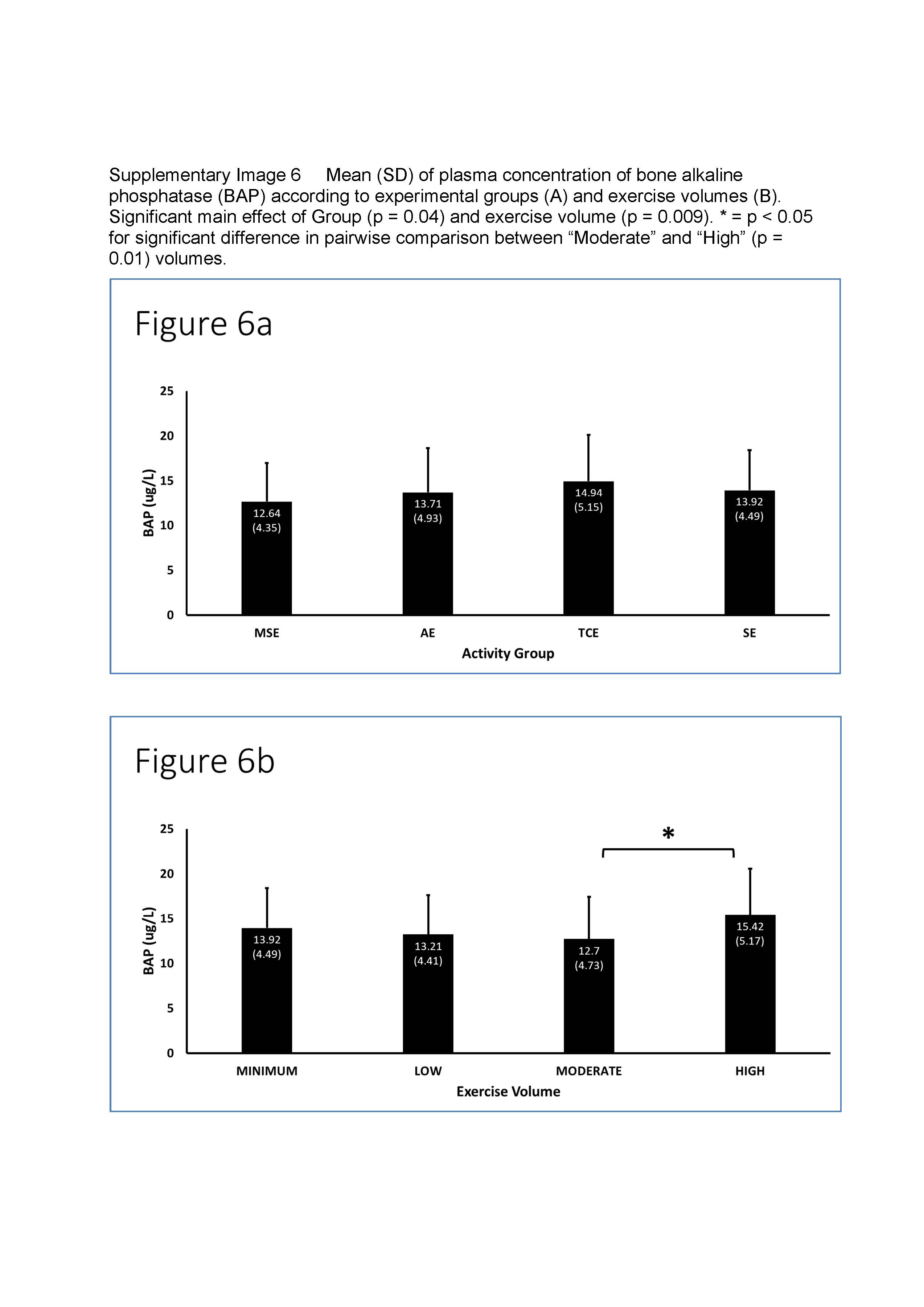

Supplement: Supplementary file 4 [file Figure6.jpg]

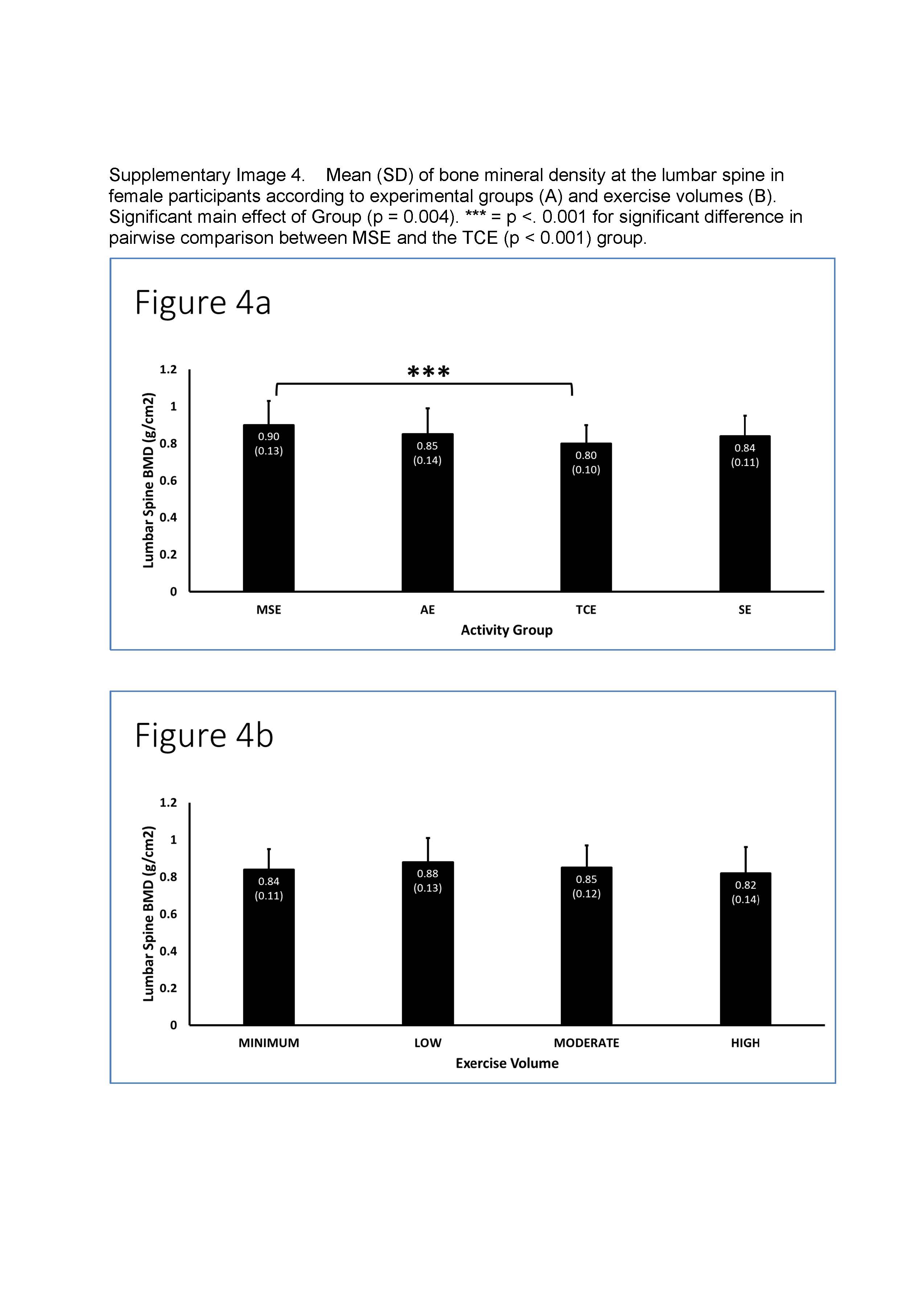

Supplement: Supplementary file 5 [file Figure4.jpg]

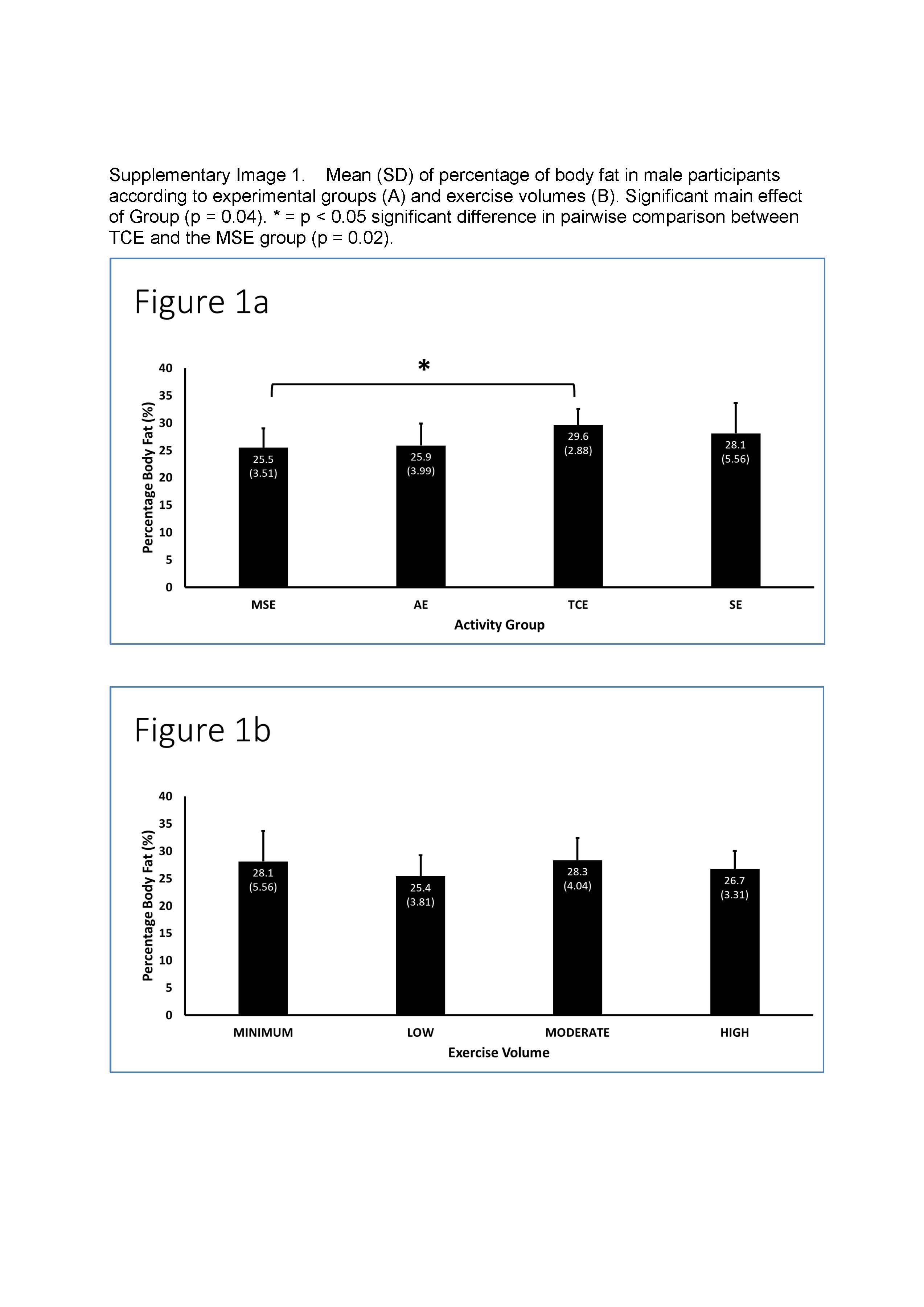

Supplement: Supplementary file 7 [file Figure1.jpg]

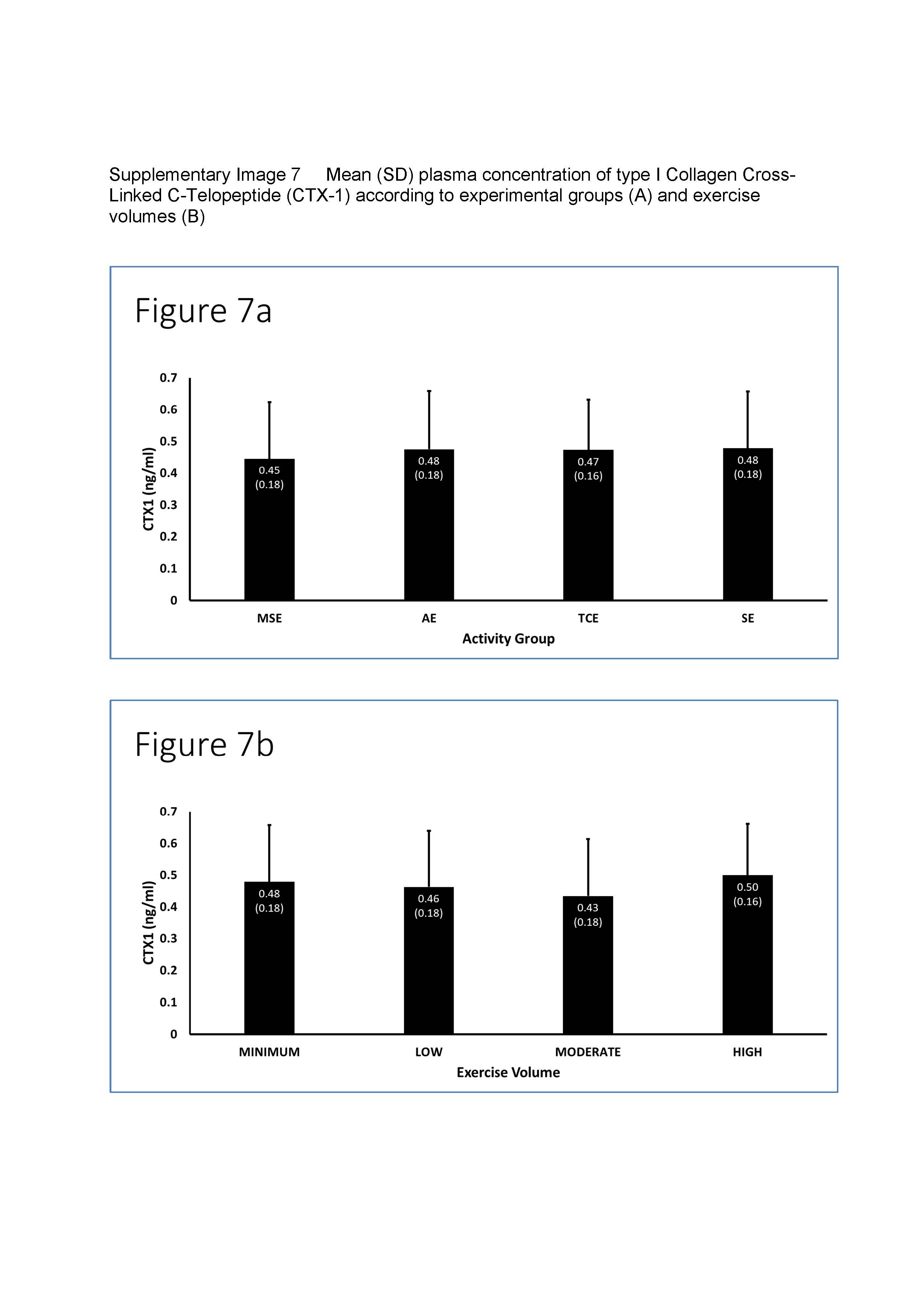

Supplement: Supplementary file 8 [file Figure7.jpg]
